# A Rare Cause of a Rare Disorder: *E. coli*-Induced Purpura Fulminans Secondary to Urinary Tract Infection

**DOI:** 10.1155/2022/9291424

**Published:** 2022-04-06

**Authors:** Jacob Lowry, Edva Noel

**Affiliations:** ^1^Department of General Surgery, Atrium Health Navicent, 777 Hemlock St, Macon, GA 31201, USA; ^2^Department of Nephrology, Temple University Hospital, 3440 N. Broad Street, Kresge West, Suite 100, PA 19004, USA

## Abstract

Purpura fulminans is a devastating thrombotic disorder infrequently encountered in medical practice and amongst the medical literature. It is a hematologic emergency in which prompt recognition and initiation of treatment are critical to mitigate its significant morbidity and mortality. Surgical evaluation is commonly required, since the debilitating skin and soft tissue necrosis often degenerate into necrotizing fasciitis, critical limb ischemia, warranting surgical interventions in either a staged or single-step approach. Purpura fulminans can be neonatal, infectious, or idiopathic. Infection-induced purpura fulminans is less common, and only a few microorganisms have been associated with this condition: *Meningococcus* spp., *Pneumococcus* spp., or *Staphylococcus* spp. This report presents a rare case of *Escherichia coli-*induced purpura fulminans. Apart from the unfortunate partial amputation of all left-hand five digits, our patient made a full recovery following effective infectious source control, supportive care with volume resuscitation, anticoagulation, and wound care.

## 1. Introduction

Purpura fulminans (PF) is a rapidly progressive thrombotic disorder that primarily affects the microvasculature of the integumentary system that quickly progresses to skin necrosis. It is associated with disseminated intravascular coagulation (DIC) with a thrombotic phenotype. Although DIC is commonly observed in the critical care setting (9-19% of patients), PF prevalence remains scarce overall [1, 2]. Neonatal, infectious, and idiopathic are the three known clinical presentations of PF. *Meningococcus* spp. and *Streptococcus* spp. account for most of the reported infectious cases [3]. PF represents a true hematologic emergency that, even if recognized and managed early, carries a mortality rate as high as 43% [4]. Current management focuses on supportive care and, in severe cases, surgical debridement or amputation secondary to serious cutaneous necrosis. We report a rare case of PF associated with *E. coli* septicemia along with a brief review of the pertinent literature regarding the pathophysiology, diagnosis, and management of PF.

## 2. Case Description

A 55-year-old African American female with medical history of hyperlipidemia, gastroesophageal reflux disease, and an anemia of unknown etiology who initially presented to an outlying hospital with complaints of left flank pain, dysuria, and malodorous urine for a few weeks. She is a nonsmoker, and her only home medications were dexlansoprazole and over-the-counter multiple vitamins. Her family history is remarkable for her father who died from heart disease and her mother and two siblings who are alive with no known health issues. Initial evaluation confirmed sepsis with a SOFA score of 1. Blood cultures and a urinalysis with reflex for culture were obtained, and empiric antibiotic with ceftriaxone was started. The remaining pertinent labs are summarized in Tables 1–3. A computed tomography scan of the abdomen and pelvis revealed a 5-mm obstructive left ureteral stone associated with hydronephrosis and perinephric stranding. A diagnosis of complicated pyelonephritis with sepsis was made. IV fluid resuscitation as per the sepsis guidelines was started. Urology service consulted and performed cystoscopy with ureteral stone removal and stent placement. Postoperatively, the patient became hypotensive requiring norepinephrine infusion; lactate increased to 6.8 mmol/L, with a SOFA score of 8. The antibiotics spectrum was broadened to piperacillin-tazobactam, and transfer to the critical care unit followed.

**Figure 1:**
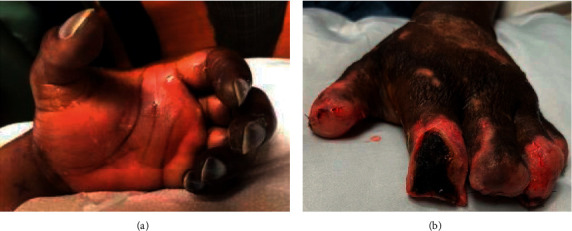
(a) The left-hand ischemia with dry gangrene of all the five digits. Also noted is the bulla on the palmar aspect of the left hand. The dorsum aspect (not shown in this figure) also had a large bulla. (b) The left hand one week postamputation of all five digits.

On admission day 2, the patient started developing purpuric skin lesions. On day 3, more acral ischemic discoloration was evident. The ischemia was worse on the left hand where a radial arterial line was inserted for accurate measurement of the blood pressure (Figure 1(a)). An arterial Doppler ultrasound confirmed a distal left radial thrombosis with preservation of the ulnar blood flow; the arterial line was discontinued, and intravenous infusion of heparin drip was started. The leukocytosis worsened to 43 × 10^3^ /*μ*L, platelets dropped to 73 × 10^3^/*μ*L, and D-dimer increased to 21.6 *μ*g/mL, but fibrinogen remained elevated to 637 mg/dL. Due to her deteriorating condition, she was transferred to our hospital for vascular surgery evaluation and higher level of care.

Upon transferring to our hospital, she no longer required vasopressors. She was afebrile but in sinus tachycardia at 115 bpm. All repeat pertinent labs including urine and blood cultures are shown in Tables 1–3. The heparin infusion and the antibiotic regimen were continued. Microbiology data from the transferring hospital confirmed pan-sensitive *E. coli* in blood and urine cultures. The symmetric acral skin lesions rapidly spread proximally within hours and evolved into large hemorrhagic bullae. The clinical picture of anemia, thrombocytopenia, and abnormal coagulation panel was consistent with DIC. The typical purpuric skin lesions in the setting of *E. coli*-sepsis and DIC led to the diagnosis of PF. At this point, hematology, general surgery, and hand surgery consultations were obtained.

**Figure 2:**
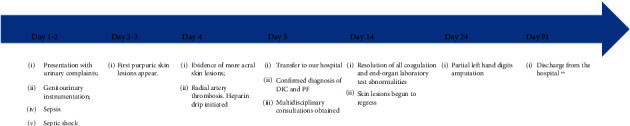
Timeline of the events summarizing the most important moments in the development and management of this patient. ∗∗ Prolonged discharge secondary to disposition issues.

She was continued on antibiotic and completed 14 days of ceftriaxone from the first negative blood culture. After 14 days of presentation, the skin lesions began to regress and did not require extensive debridement. However, the left-hand ischemia progressed to dry gangrene of all five fingers. On hospital day 24, the patient underwent partial left-hand amputation of all five digits after full demarcation of the viable tissues (Figures 1(b)). The right-hand lesions were wholly resolved, and the bilateral lower extremities significantly improved but did require prolonged daily wound care.  Figure 2 offers a timeline of the important events.

## 3. Discussion

Acute infectious PF most commonly occurs in the settings of an acute bacterial infection. *Meningococcus* spp., *Staphylococcus* spp., and *Pneumococcus* spp. in adults are the most common bacterial infections associated with PF [3]. Hyposplenia or asplenia may constitute an additional risk factor for *Pneumococcus* PF specifically. One study notes a 7-fold higher incidence of *S. pneumoniae-*induced PF than in eusplenia, despite vaccination in 35% of the patients with similar morbidity and mortality outcomes [5]. Rat models *in vivo* have also isolated one specific *Meningococcus* strain containing type IV pili that adheres to the endothelium with subsequent colony formation and microvascular thrombosis due to excessive protein C activation and a consumptive coagulopathy [6]. Type IV pili are found in 5 serotypes of *Neisseria meningitidis* A, B, C, W135, and Y, which may in part explain the petechial rash commonly observed [7]. Rare cases associated with measles virus, *Rickettsia* spp., and *Haemophilus influenzae* have also been reported in the medical literature [8]. *E. coli*-septicemia-associated PF is rare; only a handful of cases have been reported [9–11]. PF may occur because of an acute infection even in asymptomatic, afebrile, or otherwise well-appearing individuals from pediatrics to adults, but it may also transpire in the post-infectious setting. This would suggest that the severity of the disease, and therefore the quantitative load of microorganisms, is not as important as certain qualitative aspects of the microorganism that lead to the development of acute infectious FP.. In one isolated case report, pathogen-specific factors are hypothesized as a potential predisposition to PF with *E. coli* septicemia [10]. That report describes a case of *E. coli*-associated PF in a patient with a previous episode of *E. coli* bacteremia without DIC. While the source of the first bacteremia is not mentioned, the second episode is attributed to intestinal translocation favored by malnutrition following bariatric surgery. We did not run a genetic study on the pan-sensitive *E. coli* identified as the cause of the infection in our patient since this would not have affected our management. Our initial management focused on source controlled of the infection and anticoagulation.

Neonatal, idiopathic, and acute infectious purpura are the three clinical presentations described in this condition. Neonatal PF is associated with hereditary deficiency of innate anticoagulants, including protein S, Protein C, and antithrombin III. Idiopathic or chronic PF usually develops days to weeks following a bacterial or a viral infection. Although the exact pathophysiological mechanism responsible for triggering PF remains unclear, predisposing risk factors appear to be associated with either an inherited or acquired deficiency in the anticoagulation pathway. These deficiencies primarily affect protein S, protein C, and antithrombin III (AT-III).

The acquired deficiency in anticoagulant proteins in acute infectious PF appears to result from a combined effect of bacterial antigens and inflammatory cytokines [interleukin (IL)-1, IL-12, interferon gamma, and tumor necrosis factor (TNF)–*α*]. Similarly, autoimmune phenomena have been reported in the postinfectious pediatric population whereby IgG cross-reacts to protein S resulting in its severe deficiency [12]. Due to an unknown mechanism, autoimmune targeting of protein C appears to be quite rare [13]. Recombinant protein C has been successfully used in the treatment of acute infectious PF in case reports. It has also been the focus of a phase 2/3 prospective multicenter trial in patients with severe congenital protein C deficiency to treat hematological complications from the innate disease process, including PF, DIC, and other thromboembolic events [14–16]. The evidence suggests an efficacious advantage based on these results; however, significant concerns regarding hemorrhagic complications have been noted surrounding its use. Protein C recombinant therapy is an approved treatment only for congenital protein C deficiency and remains off-label in the management of PF currently.

Four key features of acute infectious PF are as follows: large purpuric skin lesions, fever, hypotension, and DIC. Any new skin lesion in the presence of DIC should raise high suspicion for PF and prompt immediate initiation of appropriate management (discussed below). The skin lesions in PF develop and evolve within hours. They are typically petechial initially then progress to large, non-blanching retiform purpura with skin necrosis and often large hemorrhagic bullae. The histopathology of these skin lesions often reveals thrombosis and extensive vascular damage: vascular congestion and dilation, endothelial necrosis, alteration of markers of endothelial integrity (CD31), and of the protein C pathway receptors (endothelial protein C receptor and thrombomodulin) [17]. It is worth underscoring that a skin biopsy is not necessary to make the diagnosis and may delay the prompt initiation of aggressive management of PF.

Our patient presented with flank pain due to ureterolithiasis and developed severe *E. coli* septicemia with shock requiring vasopressors following a urological instrumentation. Within 48 hours from the onset of sepsis, the first skin lesions appeared as a petechial rash. At that time, the patient was already exhibiting findings of DIC characterized by an elevated prothrombin time (PT) and aPTT, elevated D-dimer, and thrombocytopenia. In PF, the peripheral smear often shows a pattern of microangiopathic hemolytic anemia with schistocytes and helmet cells. Our patient's smear did not show schistocytes, but a DIC score per ISTH criteria was 5, which was high. The skin lesions rapidly evolved into large hemorrhagic bullae within 48 hours of appearance. TTP, HSP, and postinfectious thrombocytopenic purpura are all secondary skin lesions like PF but do not match the severity of the necrosis seen in our patient and therefore were ruled out. The case reported by Morales et al. is very similar to our case both in the presentation and the management [9]. Both cases highlight the importance of nurturing a high clinical suspicion for PF in the presence of purpuric skin lesions in septic patients even in the absence of a classic picture of DIC. In PF, the debilitating skin lesions often require extensive surgical debridement. In severe cases, limb amputation is necessary, resulting in significant disability, and in the worst cases, PF results in death [18].

For our patient, a multidisciplinary team composed of hematologists, surgeons, and wound care was assembled. Effective empiric antibiotic therapy was started along with aggressive volume resuscitation 30 mL/kg as per the sepsis guidelines. Initiation of systemic anticoagulation, preferably with heparin drip, has the potential to lessen the skin necrosis process, and the thrombotic burden in the medium-sized vessels is also pivotal in the management [19, 20]. Our patient was started on a continuous infusion of heparin that was monitored via aPTT with a target therapeutic range between 46 s and 70s. When a therapeutic aPPT level is difficult to attain despite a high dose of heparin, resistance to heparin should be considered. This situation, if it arises, should raise concerns for acquired protein C and ATIII deficiency. In such a case, co-administration of FFP or antithrombin III concentrate with protein C is reasonable [21]. We were able to easily achieve a therapeutic aPPT level in our patient. Hyperbaric oxygen (HBO) therapy has been used as an adjunctive treatment for chronic nonhealing wounds to promote healing [22]. Our patient's wounds progressed favorably with local care; they did not require extensive debridement; thus, we did not feel that HBO was necessary. In the medical literature, there is no mention of the type and duration of anticoagulation therapy for thrombosis in patients with PF without protein S or C or AT-III deficiency. Based on the current deep venous thrombosis management guidelines, our patient was continued on an oral direct thrombin inhibitor for an anticipated six months.

## 4. Conclusion

PF is an acute thrombotic hematologic emergency. Unfortunately, research has yet to establish the exact pathophysiological mechanism triggering its development. Current risk factors include infection by a select few microorganisms on the background of congenital or acquired anticoagulant deficiencies. In addition to its low overall incidence, diagnosis of PF, regardless of etiology, can prove difficult, especially in the critical care setting where skin lesions are frequent. The bacteria mentioned in this report demonstrate that the few encapsulated microorganisms with a well-documented link with PF may not be unique to this condition. Therefore, early recognition and treatment remain crucial to mitigate the devastating morbidity and mortality associated with this morbid complication. The mainstay of treatment currently is primarily supportive, including source control of infections, therapeutic anticoagulation, or any other justified therapies on an individualized basis.

## Figures and Tables

**Table 1:** Laboratory values upon initial presentation to our facility. Of note, patient remained on continuous heparin infusion during time of lab results.

Lab tests	Hospital day 1 (outlying hospital)	Hospital day 1 (this hospital)	Hospital day 5 (this hospital)	Units (normal range)
Hemoglobin	12.8	11.5	10.6	11.6-15 g/dL
Hematocrit	39.3	35.6	33.7	35.5-44.9%
White blood cell count	13.8 with 13% bands	55.96	19.9	3.4-10.8 K/mm^3^
Platelets	203 (nadir 73)	92	288	150-379 K/mm^3^
Sodium	140	143	137	135-145 mmol/L
Potassium	3.6	3.9	4.7	3.5-5 mmol/L
Chloride	110	111	104	95-105 mmol/L
Bicarbonate	24	24	24	23-29 mmol/L
Blood urea nitrogen	20	21	8	5-20 mg/dL
Creatinine	1.5	0.66	0.67	0.61.2 mg/dL
Albumin	4	2.7	2.9	3.5-4.8 g/dL
Aspartate aminotransferase	26	227	124	0-40 U/L
Alanine transferase	20	114	74	0-32 U/L
Lactate	6.8	1.5	2.2	Mmol/L
Creatinine phosphokinase	∗	5976	1002	22-198 U/L
Activated partial thromboplastin time (PTT)	∗	55.9 (on heparin drip)	65.5 (on heparin drip)	25-35 seconds
Prothrombin time (PT)	∗	14.7	14.3	Seconds
International normalized ratio (INR)	∗	1.2	1.15	N/a
D-dimer	∗	13.95	3.66	<0.5 *μ*g/mL
Total bilirubin	∗	0.5	0.2	0.0-1.2 mg/dL
Fibrinogen	∗	622	619	200-400 mg/dL
Haptoglobin	∗	∗	284	41-165 mg/dL
Antinuclear antibody (ANA) screen	∗	∗	Positive (RNP ab)	0.2 - 0.9 AU/mL
p/c-Antineutrophil cytoplasmic antibodies (ANCA)	∗	∗	0.2	<0.4 (negative)
Complement factors 3, 4	∗	∗	116, 21	82-193 mg/dL, 15-57 mg/dL
Protein S, functional:	∗	∗	79%	60-123%
Protein C, functional:	∗	∗	174%	70-130%

∗Indicates lab value either not drawn or not available due to lab error.

**Table 2:** Urinalysis upon initial presentation to outside facility on April 19, 2021.

Urinalysis results	
Leukocyte esterase	Trace
WBC count	19/hpf
Nitrite	Negative
Bacteria	Trace

**Table 3:** Microscopic analysis of urine from initial sample at outside facility.

Microbiology and serology	
Urine culture	*+E. coli*
Blood culture	*+E. coli*
Hepatitis panel	Negative
HIV serology	Negative
